# Youngest presenting patient with dystonia 24 and review of the literature

**DOI:** 10.1002/ccr3.1671

**Published:** 2018-09-15

**Authors:** Sarah Nelin, Richard Hussey, Brian M. Faux, Luis Rohena

**Affiliations:** ^1^ Brooke Army Medical Center San Antonio TX USA

**Keywords:** *ANO3*, DYT24, whole‐exome sequencing

## Abstract

Dystonia 24 was first reported in 2000 as an autosomal dominant cause of dystonia caused by variants in the *ANO3* gene. Although many adults have been described with dystonia 24, since 2014, an increasing number of children have also been reported. Dystonia 24 should also be considered in the differential of a child with unexplained dystonia.

## 1 INTRODUCTION

Dystonia 24 (DYT24) is a recently classified cause of craniocervical dystonia which affects the *ANO3* gene and is associated with autosomal dominant inheritance. To date, 25 cases have been identified and studied with 11 known *ANO3* gene variants. Previously identified variants have been predominantly adult onset disease with phenotypic manifestations including torticollis, dysphonia, blepharospasm, tremor, and myoclonus. Here we present the case of a child with symptoms of developmental regression, dystonia, and Parkinsonism with a protracted diagnostic course in an effort to expand the phenotypic presentation of DYT24. Whole‐exome sequencing (WES) identified a heterozygous, de novo variant c.1819A>T; p.I607F in exon 17 of the *ANO3* gene predicting the diagnosis of DYT24. To our knowledge, this is the earliest presentation of symptoms in Dystonia 24 after review of the reported literature. Next generation sequencing tests have led to exponential increase in genetic diagnosis of various forms of dystonia, and our patient provides further evidence that Dystonia 24 should be considered in the differential of a child with unexplained dystonia.

Dystonia (DYT) is a movement disorder generally characterized by sustained or intermittent muscle contractions with abnormal postures, abnormal movements, or both.^1^ Dystonia is typically classified based on clinical features and etiology, with clinical classification focusing on age at symptom onset, focus of onset, symptom distribution, symptom progression, response to treatment, and other symptoms present.^2^ Cervical dystonia is often the most common focal dystonia seen in clinics.^3^ Most DYTs are classified etiologically according to the genes involved vs other environmental or disease processes that may cause the specific dystonia. Genetic DYTs are numbered according to the order of identification.

DYT24 (OMIM #615034) was first identified in 2000 by Munchau et al.^4^ This study identified the index family with 5 individuals affected by tremulous craniocervical dystonia and described an autosomal dominant inheritance pattern. The study ruled out associations with DYT1, 6 or 7. The gene associated with this form of dystonia was identified in 2012 in a study which found 3 unrelated individuals with similar craniocervical dystonia.^3^ The phenotype was expanded in 2014 with affected patients demonstrating symptoms as young as 3‐4 years of age, however, with the majority of symptom onset occurring in adulthood.^5^ Clinical features include predominantly craniocervical dystonia including torticollis, laryngeal dysphonia, adductor dysphonia, oromandibular dystonia, and blepharospasm. All cases were also associated with tremor affecting the head, upper limb, or posture. Some cases are associated with upper limb dystonia or myoclonus.^5^

Recent research has also been able to further characterize the protein encoded by the *ANO3* gene, also known as, anoctamin 3. This protein belongs to a family of 10 anoctamin proteins that function as calcium‐activated chloride channels of which the most is known about ANO1 and ANO2.^6^ ANO1 and ANO2 are known to function at the cell surface with roles in sensory transduction, secretion at the epithelial membrane, and smooth muscle contraction.^7^ It has been shown that ANO3 is not expressed at the cell surface but is an intracellular transmembrane protein likely working at the endoplasmic reticulum.^3^, ^7^ The ANO3 protein was highly expressed in brain tissue, specifically the striatum but also in the neocortex, hippocampus, and amygdala.^3^ Gene expression increases during development from the fetal stage to adolescence.^8^ Knowing the function of this protein as part of neuronal excitability with a location in the brain can potentially explain its role in dystonia; however, much remains unknown.

Earliest identified missense variants in the *ANO3* gene include: c.1480A>T; pR494W and c.1470G>C; p.W490C in exon 15, as well as, c.2053A>G; p.S685G, c.2586G>T; p.K862N and c.‐190C>T.^3^ A more recent study expanded the number of identified missense variants: c.2497A>G; p.I833V, c.2917G>C; p.G973R, c.704A>G; p.Y235C, c.767A>G; p.N256S, and c.2678C>T; p.P893L (latter three found in healthy controls).^9^ Another variant was further identified c.2540A>G; p.Y847C.^1^ In 2016, a novel c.1969G>A variant in *ANO3* was discovered in a family with stereotypic DYT24 symptoms.^10^, ^11^ identified one more missense variant which arose de novo as in our patient: c.1528G>A; p.G510L. Their study also identified the previously seen c.1969G>A; p.A657T variant in a different patient. Both of these final gene variants are associated with a phenotypic difference from previously described cases in that they had lower limb involvement.^11^

The content of this manuscript is not considered research at our institution and instead falls in the realm of routine clinical care.

## 2 STUDY CASE

Our patient is an 8 yo M referred to genetics secondary to dystonia, Parkinsonism, and developmental regression. He was born to nonconsanguineous parents via induced spontaneous vaginal delivery at 39 weeks secondary to LGA status. Prenatal course was complicated by preterm labor starting at 33 weeks, mother received antenatal steroids, and an amniocentesis was performed to assess fetal lung maturity. History of maternal tobacco use during pregnancy (5 cigarettes/day), however no other known prenatal exposures. Delivery was uncomplicated with infant weighing 4025 g (92^nd^ centile) length of 55 cm (90th centile) and Head Circumference of 39 cm (97th centile) at birth. Postnatal hospital stay was uncomplicated, and patient was discharged home with mother after one night. Earliest parental concern was at 2 months of age with unprovoked left leg twitching which spontaneously resolved. Early development was uncomplicated until about 18 months of age. Patient presented with ataxia and difficulty walking. He attained the skill of walking at 14‐15 months of age; however, around 18 months of age was unable to walk on his own anymore. Physical examination at this time was remarkable for macrocephaly, hypotonia, and questionable decrease in upper extremity reflexes. The patient also developed expressive language skill regression, going from a vocabulary of 30‐50 words with the ability to combine 3 word sentences at 22 months of age to nonverbal at 2 years of age. Modest increase in motor skills and verbal skills for 3 weeks around his 2nd birthday; however, these skills were again lost. Patient continued to have progressive worsening of gross motor skills with development of on and off tremors over next several months (of note, fine motor skills relatively preserved until this point).

Physical examination completed at approximately 32 months of age remarkable for bradykinesia, global rigidity, central hypotonia, decreased strength, Parkinsonism, and myoclonus. Patient started physical therapy, speech therapy, and occupational therapy. Trial of carbidopa/levodopa started with initial improvement in symptoms, however, patient unable to tolerate medication. At 3 years of age, patient was using a wheelchair for long‐distance ambulation with loss of ability to crawl on 4 points, instead crawling commando style and rolling towards desired objects. He was no longer able to self‐feed and began to require soft food diet secondary to choking on food. The patient also developed painful leg spasms that were worse at night. Trial of baclofen and clonazepam for painful spasms/sleep with improvement in pain symptoms as well as motor skills, however, over time efficacy dwindled despite multiple titrations in medication. Gastrostomy tube was placed at approximately 3 1/2 years of age secondary to failed swallow study and weight loss. At 4 years, the patient remained unable to walk (low‐crawling for ambulation), had a vocabulary of <10 words (although able to shake head yes/no, say ABCs and count), and continued to have central hypotonia, myoclonus, and painful spasms. Trial of trihexyphenidyl was added to the medication regimen with improvement in symptoms with ability to crawl on 4 points, bear weight, and cruise. At 5 years of age, baclofen was discontinued secondary to dwindling efficacy and diazepam was started. Throughout this time, there was an overall slow progression of symptoms with acute worsening when the patient was ill. At 5 1/2 years of age, botulinum toxin injections were initiated which improved symptoms for 2‐3 months at a time. Carbidopa/levodopa was restarted with benefit. At 8 years of age, patient's symptoms continue to worsen with generalized dystonia and global developmental delay. Current medication regimen includes carbidopa/levodopa, baclofen, diazepam, clonazepam, and botulinum toxin injections. His current height is 48 cm (43rd centile), current weight is 23.3 kg (24th centile), and current head circumference is 56 cm (>98th centile).

Multiple laboratory and imaging studies were completed. Urine mucopolysaccharide screening negative. Normal CBC, LFTs, chemistry, creatinine kinase, TSH, lactate and ammonia levels. Normal pyruvate, carnitine, plasma and urine amino acids. Normal ceruloplasmin and copper levels. Initial very long‐chain fatty acid analysis showed moderate elevation of C26 and C26/C22 ratio with minimal elevation in C24 and C22; however, repeat analysis was normal. Normal CSF neopterin, tetrahydrobiopterin, homovanillic acid, 3‐O‐methyldopa, and 5‐hydroxyindoleacetic acid with otherwise normal CSF cell counts, protein, and glucose. Negative spinocerebellar ataxia type 2 and 3 genetic testing, as well as negative *PARK2* testing (early‐onset Parkinsonism). Dopa‐responsive dystonia evaluation reported variant of unknown significance that was likely benign in the TH sequencing variant: IVS12 + 9 C>T. Normal glutaryl‐coA dehydrogenase enzyme activity in fibroblasts. Microarray completed at 6 years old was negative. MRI at 1 year reported nonspecific white matter changes, a small arachnoid cyst in the posterior fossa, J‐shaped sella, and mild tonsillar ectopy. Repeat MRI with MR spectroscopy completed at 2 years of age showed nonspecific white matter changes without spectroscopic abnormality. CXR and skeletal surveys were normal. Multiple EEGs were completed and were normal. Hearing and vision screening were also normal.

## 3 MOLECULAR STUDIES

Untargeted whole exome sequencing was performed on the patient and his unaffected parents. Average coverage was 96.46%. Our patient was found to be heterozygous for a de novo missense variant in *ANO3* c.1819A>T which predicts the corresponding protein change of p.I607F. The predicted inheritance pattern for this gene is autosomal dominant. Co‐segregation analysis of this alteration revealed that both unaffected parents do not carry this alteration. This alteration was not seen in 6478 healthy individuals in the NHLBI exome sequencing project. Allele frequency data for this nucleotide position are not currently available from the 1000 Genomes Project or ExAC, and the alteration is not currently listed in the Database of Single Nucleotide Polymorphisms (dbSNP). The p.I607 amino acid is completely conserved in available vertebrate species. The p.I607F alteration is predicted to be probably damaging by Polyphen and deleterious by SIFT in silico analyses.

## 4 DISCUSSION

The term dystonia covers a broad category of involuntary movement disorders that can be associated with posturing or abnormal movements. To date, there are 23 identified primary dystonia syndromes and, of these 23 DYTs, twelve have onset in childhood, adolescence, or young adulthood prior to the age of 30 (termed early‐onset): DYT1, DYT2, DYT4, DYT5, DYT6, DYT11, DYT13, DYT16, DYT17, DYT23, DYT24, DYT25.^2^, ^12^ The patient presented here carries a variant in the *ANO3* gene which has previously been known to cause DYT24 which is a predominantly adult onset (although cases as early as 3‐4 years old) cause of tremor and craniocervical dystonia.^11^ No other variants were reported to be associated with the patient's phenotype meaning our patient is critical in expanding the phenotype for DYT24 with earliest age of onset, lower leg involvement, Parkinsonism, and developmental regression. A comparison of known features and variants from previous reports with the patient described here can be seen in Table 1. On review, it seems that earlier age of onset is associated with a more severe form of disease that includes extensive developmental delay, gastrostomy‐tube dependence, infantile Parkinsonism, and generalized dystonia as compared to patient's affected solely with movement symptoms.

**Table 1:** Gene variant and clinical phenotype

cDNA variant	Protein Alteration	Clinical phenotype	Decade of onset	Reference
c.161C>T	p.Thr54Ile	Diagnosed as familial essential tremor	Unknown	3
c.2053A>G	p.Ser685Gly	Autosomal dominant cervical dystonia, laryngeal dystonia, dystonic tremor of upper extremities, myoclonus	1st	3
c.2586G>T	p.Lys862.Asn	Cervical and oromandibular dystonia	2nd‐4th	3
c.‐190C>T		Cervical dystonia, upper extremity tremor, myoclonic jerks	2nd	3
c.2497A>G	p.Ile833Val	Cervical dystonia, dystonic head tremor	5th	9
c.2917G>C	p.Gly973Arg	Blepharospasm, oromandibular dystonia	7th	9
c.1528G>A	p.Glu510Lys	Craniocervical, bulbar, laryngeal, truncal and lower extremity dystonia, upper extremity tremor, and myoclonic jerks	2nd	11
c.1969G>A	p.Ala657Thr	Generalized dystonia, myoclonic jerks of lower extremities and trunk, upper extremity tremor	2nd	11
c.1480A>T	p.Arg494Trp	Cervical torticollis, laryngeal and oromandibular dystonia, blepharospasm, mild upper extremity dystonia, head and upper extremity tremors	2nd‐5th	3
c.1470G>C	p.Trp490Cys	Cervical torticollis, laryngeal and oromandibular dystonia, blepharospasm, mild upper extremity dystonia, head and upper extremity tremors, myoclonus	1st‐3rd	3
c.2540A>G	p.Tyr847Cys	Craniocervical dystonia, blepharospasm, dysphonia, head and upper extremity tremors	4th	1
c.1819A>T	p.Ile607Phe	Generalized dystonia, infantile Parkinsonism, bradykinesia, global developmental delay, myoclonus	1st	Our patient

In addition, the patient presented here had a protracted diagnostic course with WES revealing the diagnosis of *ANO3* gene variant and dystonia 24. If WES had not been available, this could lead to misdiagnosis, as well as missed opportunities to discover appropriate treatments. Diagnosis in genetic syndromes, especially those with vague and varied presentations, is difficult to make on clinical grounds alone. These diagnoses are also more difficult secondary to the genetic heterogeneity that is seen in many diseases. The diagnostic yield of WES is higher in patients with heterogeneous diseases that include blindness, deafness, and (as in our patient) movement disorders.^13^ Determining specific diagnoses is useful to determine etiologies, prognosis, inheritance patterns, and effective treatments. Much of the extensive diagnostic work up on this patient could have been prevented if WES was considered earlier in his course. We recommend WES in patients with unknown explanations for their dystonia. A recently published systematic review by van Egmond et al outlined a diagnostic algorithm for children and adolescents presenting with dystonia. This algorithm includes next‐generation sequencing testing earlier rather than focusing on pattern recognition for known dystonia syndromes as a result of the clinical heterogeneity seen in many dystonia syndromes.^14^ Although DYT24 has been predominantly an adult‐onset disorder, it should remain a part of the differential diagnosis of a child with dystonia.

Treatment of dystonia 24 in our patient has proven to be difficult with multiple medications showing at least some benefit initially with waning efficacy after prolonged use and other medications causing side effects that limited their uses. Regardless of underlying diagnosis, the current treatment of dystonia is mainly symptomatic control with three broad categories of treatment including oral medications, chemodenervation (botulinum toxin injections), and surgical treatments to provide deep brain stimulation. The decision regarding treatment modality focuses on patient age, severity of disease, distribution of disease, and co‐existing morbidities.^2^, ^15^ WES has allowed advancements in diagnosis which has led to advances in understanding disease causing etiologies. Future research should aim at advances in treatment of dystonia in general, as well as if there is utility in diagnosis specific treatments for primary dystonias.

## AUTHORSHIP

SN reviewed patient clinical charts, labs, radiology studies Department of the Air Force.

## CONFLICT OF INTEREST

All authors have no conflicts of interest to disclose and there was no financial support.
